# Transcriptomic associations and biomarkers in ANCA‑associated Glomerulonephritis and IgG4‑related Disease: A bioinformatics and machine learning study

**DOI:** 10.1371/journal.pone.0358953

**Published:** 2026-09-24

**Authors:** Xu Li, Yufeng Qiao

**Affiliations:** 1 The Nephrology Department of Shanxi Provincial People’s Hospital, Shanxi Medical University, Taiyuan, China; 2 The Nephrology Department of Shanxi Provincial People’s Hospital, Taiyuan, China; Versiti Blood Research Institute, UNITED STATES OF AMERICA

## Abstract

**Introduction:**

Anti-neutrophil cytoplasmic antibody-associated glomerulonephritis (ANCA-GN) and immunoglobulin G4-related disease (IgG4-RD) are rare autoimmune conditions. Although case reports suggest potential clinical and pathophysiological overlaps, the cross-tissue transcriptional associations between these diseases remain largely unexplored.

**Methods:**

We retrieved the ANCA-GN glomerular dataset (GSE104948) and the IgG4-RD labial salivary gland dataset (GSE40568) from the Gene Expression Omnibus. Differentially expressed genes (DEGs) in ANCA-GN were screened via protein–protein interaction networks, least absolute shrinkage and selection operator regression, and the Boruta algorithm. Given tissue heterogeneity and small IgG4-RD sample size, we used expression trend consistency (log-fold change direction) rather than differential validation to explore shared transcriptional features. Receiver operating characteristic curves assessed discriminative potential. Pathway associations were explored using gene set enrichment analysis, and immune infiltration was evaluated with CIBERSORT, single-sample gene set enrichment analysis, and MCPcounter. Transcription factor–mRNA–microRNA networks were also constructed.

**Results:**

We identified 305 DEGs (209 upregulated, 96 downregulated) in ANCA-GN, enriched in immune/inflammatory pathways including phosphatidylinositol 3-kinase–Akt and mitogen-activated protein kinase signaling. After machine learning and cross‑trend comparison with IgG4‑RD, ITGAM and CXCL1 were identified as candidate cross‑tissue markers, with ITGAM demonstrating robust predictive performance in both internal and external validation (AUC = 0.921, AUC = 0.844, respectively). CXCL1 showed good internal discrimination (AUC = 0.937) but substantially lower external validity (AUC = 0.622), suggesting limited generalizability. ITGAM therefore emerges as a more reliable cross‑disease biomarker candidate, whereas CXCL1 requires further validation. Both diseases showed increased B‑cell, T‑cell, and monocyte infiltration, but effector profiles differed: neutrophils dominated in ANCA‑GN, while plasma cells/M2 macrophages dominated in IgG4‑RD. Regulatory analysis suggested SPI1 may regulate ITGAM, and CEBPD, NFKB1, and RELA may drive CXCL1 expression under inflammation.

**Conclusion:**

This study systematically revealed shared differential expression trends and immune‑related pathway enrichment features between ANCA‑GN and IgG4‑RD at the transcriptomic level, and identified ITGAM and CXCL1 as potential cross‑tissue candidate biomarkers, offering novel insights into the transcriptomic‑level cross‑tissue associations between the two diseases. However, these findings are entirely derived from bioinformatics predictions and require further experimental validation. The low AUC value of CXCL1 in the external validation underscores this need.

## 1. Introduction

ANCA-associated vasculitis (AAV) is a group of rare, heterogeneous autoimmune diseases characterized by necrotizing vasculitis of small and medium-sized vessels and the production of anti-neutrophil cytoplasmic antibodies (ANCA). The clinical presentation of AAV typically involves multiple organ systems. As the kidney is an organ highly sensitive to perfusion, the most common and severe manifestation of AAV is rapidly progressive glomerulonephritis (RPGN). This presents with acute decline in renal function, proteinuria, microscopic hematuria, and hypertension, and is termed ANCA-associated glomerulonephritis (ANCA-GN) [1]. IgG4-related disease (IgG4-RD) is an immune-mediated, multisystem, fibrotic, inflammatory disorder characterized by tumor-like lesions, infiltration of IgG4 + plasma cells, layered fibrosis, and occlusive venulitis. Its imaging manifestations reveal multiorgan fibrosis, predominantly involving the head and neck salivary glands (especially the submandibular gland), liver, kidneys, and lungs [2]. IgG4-related kidney disease (IgG4-RKD) is an umbrella term encompassing all forms of IgG4-related kidney disease. The most common manifestations are IgG4-related tubulointerstitial nephritis (IgG4-TIN) and IgG4-related membranous glomerulonephritis (IgG4-MGN). Clinically, IgG4-RKD may present as acute or chronic kidney injury, nephrotic syndrome, space-occupying lesions, and obstruction [3].

In 2017, François-Xavier Danlos et al. reported findings on ANCA-associated vasculitis (AAV)/IgG4-related disease (IgG4-RD) overlapped syndrome, suggesting potential overlapping mechanisms in the pathogenic pathways of AAV and IgG4-RD [4]. Increasing case reports have documented the overlap between ANCA-GN and IgG4-RKD. However, differing perspectives persist regarding whether these represent an overlapping syndrome or distinct mechanisms within a single disease entity [5–10]. In current clinical practice, rituximab serves as first-line therapy for this overlap syndrome, providing symptom relief for both conditions [11]. However, the efficacy of rituximab—a component of treatment regimens for various autoimmune diseases—does not necessarily indicate a shared pathogenic mechanism between the two conditions. Therefore, identifying pathways or biomarkers that are common to both diseases could aid in optimizing therapeutic strategies for overlap syndromes and ultimately improving patient outcomes.

The advancement of bioinformatics provides a novel approach and perspective for investigating the shared genetic mechanisms underlying ANCA-GN and IgG4-RKD. Since no IgG4-RKD dataset exists in the GEO database, we selected labial salivary gland (LSG) tissue from patients with IgG4-related sialadenitis for renal tissue to elucidate the shared cross‑tissue transcriptomic signals between ANCA-GN and IgG4-RD. We screened for differentially expressed genes (DEGs) in ANCA‑GN, constructed protein‑protein interaction (PPI) networks, and identified key candidate genes using machine learning algorithms. These findings were further validated in IgG4‑RD to explore biomarkers shared between the two diseases. Additionally, we examined the correlations between these genes and various immune cell types, and constructed a TF–mRNA–miRNA regulatory network. Therefore, we propose that ANCA-GN and IgG4-RD share common cross-tissue transcriptomic features and candidate biomarkers.

## 2. Materials and methods

### 2.1 Data collection and processing

**Fig 1:**
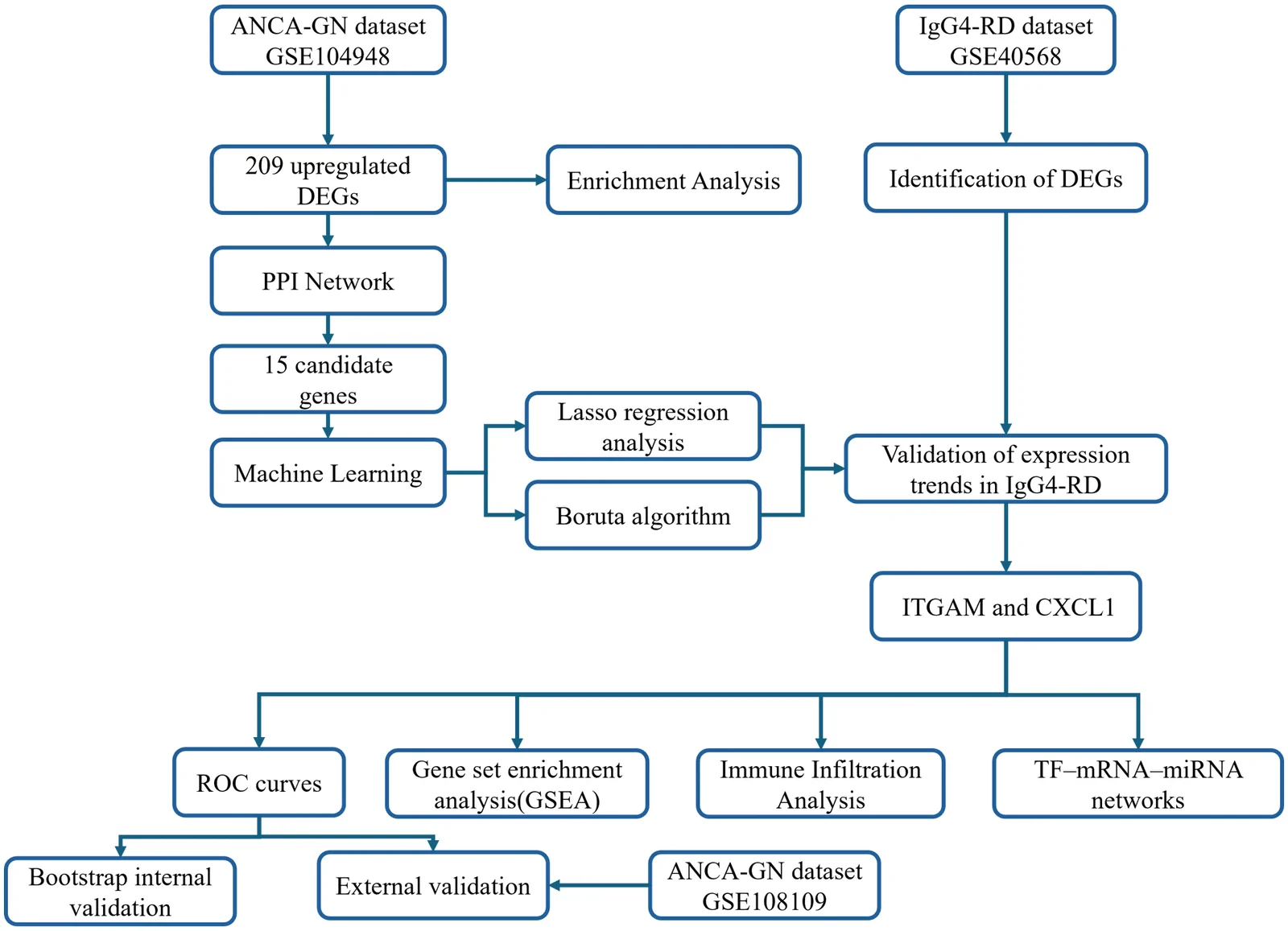
Overall workflow of this study.

We searched the Gene Expression Omnibus (GEO,https://www.ncbi.nlm.nih.gov/geo) of the National Center for Biotechnology Information (NCBI) for relevant datasets using the keywords “ANCA-associated vasculitis-related glomerulonephritis(ANCA-GN)” and “IgG4-related disease (IgG4-RD)”. Our screening criteria required datasets with ≥8 samples and inclusion of both disease and control groups. For ANCA-GN, we selected GSE104948 based on the GPL22945 platform, comprising 22 ANCA-GN patients and 18 healthy controls. Tissue samples originated from glomeruli of the patients and living donors. For IgG4-RD, we selected GSE40568 based on the GPL570 platform, including 5 IgG4-RD patients and 3 healthy controls. Tissue sources were labial salivary gland (LSG) samples from IgG4-RD patients and healthy individuals. In addition, for external validation, we selected the independent ANCA‑GN dataset GSE108109, which was generated using the GPL19983 platform and comprises 15 ANCA-GN patients and 6 healthy controls. The tissue samples in this dataset were derived from glomeruli. The overall workflow is summarized in Fig 1.

Schematic workflow of this study: (1) Data retrieval from GEO (GSE104948, ANCA‑GN; GSE40568, IgG4‑RD) and batch correction; (2) DEG identification (limma, |log_2_FC| > 1, adj.P < 0.05); (3) Functional enrichment and PPI network construction; (4) Machine learning (LASSO + Boruta) to select candidates; (5) Cross‑disease trend validation by log_2_FC direction; (6) Final characterisation including ROC(Bootstrap internal validation and external validation), GSEA, immune infiltration, and TF–miRNA network. All analyses were done in R 4.5.1.

### 2.2 Identification of DEGs

We performed data correction on the obtained expression matrix and annotated gene names using R software (version 4.5.1). As GSE104948 and GSE40568 originated from distinct platforms and batches, we applied the SVA package (version 3.52.0) to estimate and correct for batch effects, aiming to minimize their confounding influence on the differential expression analysis. We used the limma package (version 3.60.3) to perform differential expression analysis between the two groups, applying an empirical Bayesian moderated t‑statistic. To account for multiple hypothesis testing across the genome, raw P‑values were adjusted using the Benjamini–Hochberg (BH) method to control the false discovery rate (FDR). Differentially expressed genes were defined by an adjusted P‑value < 0.05 and |log_2_FC| > 1. Finally, gene correlation heatmaps and volcano plots were generated using R (version 4.5.1) to visualize DEGs.

### 2.3 Functional enrichment analysis

Given the limited sample size of the IgG4‑RD dataset (GSE40568), the enrichment analysis results may be statistically underpowered; consequently, we restricted our functional annotation analysis to the differentially expressed genes (DEGs) derived from the ANCA‑GN dataset (GSE104948).The “clusterProfiler” package was employed to perform Gene Ontology (GO) annotation and Kyoto Encyclopedia of Genes and Genomes (KEGG) pathway enrichment analysis on the genes of GSE104948, providing further insights into their biological functions and physiological significance. Data with adjusted p < 0.05 were considered statistically significant and indicated as significantly enriched. Enriched pathways were visually represented using bar charts.

### 2.4 Construction of Protein–Protein Interaction (PPI) Network

Protein-protein interaction networks are complex networks formed by interactions between individual proteins. By constructing PPI networks from screened genes, we can understand gene interactions and identify highly relevant genes. The genes of GSE104948 obtained after intersection were imported into the STRING (https://cn.string-db.org). With a confidence score threshold of ≥0.7, a PPI network diagram was constructed to evaluate interactions between genes. Subsequently, Cytoscape software was used to visualize the results obtained from the STRING database. We used the Cytoscape plugin HubbaTable to screen for the top 15 genes based on the Maximal Clique Centrality (MCC) derived from the topological algorithm.

### 2.5 Identification of candidate genes via machine learning algorithms

We used LASSO regression with 10‑fold cross‑validation to select the optimal λ and identify candidate genes minimizing cross‑validation error. The Boruta algorithm was then applied to generate shadow features via random forest; after 1,000 iterations, Z‑score comparisons of importance between true and shadow features were used to select disease‑associated genes. The intersection of the two methods was defined as the final candidate set to reduce false positives from single‑algorithm biases. Candidate genes that were shared between the two diseases were identified by taking the intersection of the results from these two methods.

### 2.6 Validation of candidate genes in the IgG4‑RD dataset

Given the limited sample size of the IgG4‑RD dataset, the statistical power of its differential expression analysis is constrained, rendering it challenging to obtain robust significant results. Therefore, in this study, we based our validation strategy primarily on the consistency of expression trends rather than on statistical significance. Specifically, we mapped the differentially expressed genes (DEGs) identified in the ANCA‑GN dataset to the differential expression results of the IgG4‑RD dataset and extracted the log_2_FC values for each corresponding gene. A log_2_FC > 0 was used as the cutoff to define genes that were also upregulated in IgG4‑RD, which were considered to exhibit a consistent upregulation trend across both diseases, whereas a log_2_FC < 0 indicated an opposite trend. This approach does not require the differential expression in IgG4‑RD to reach statistical significance; instead, it serves as a preliminary assessment of whether the expression trends are conserved across the two diseases.

### 2.7 Validation of candidate genes using ROC curves

To evaluate the predictive value of candidate genes in ANCA‑GN, we performed internal validation via bootstrap resampling (200 iterations) on the GSE104948 dataset and calculated the AUC with its 95% confidence interval (CI). Subsequently, to assess the generalizability of these biomarkers across different ANCA‑GN cohorts, we selected the independent GSE108109 dataset for external validation and computed the corresponding AUC and CI. All receiver operating characteristic (ROC) curves were generated using the pROC package in R.

### 2.8 Gene set enrichment analysis (GSEA)

Gene Set Enrichment Analysis (GSEA) is a pathway enrichment method based on genome‑wide expression profiles. The analysis employs gene set permutation testing (1,000 permutations) to identify significantly enriched pathways, using normalized enrichment scores (NES) and an FDR < 0.05 as criteria. Using the GSEABase R package, we performed single‑gene GSEA on the differentially expressed genes (DEGs) identified in the ANCA‑GN dataset, separately in the GSE104948 and GSE40568 cohorts. For each target gene, we computed its expression correlation with all other genes in the same dataset, ranked the genes by correlation coefficient in descending order, and conducted enrichment analysis against the KEGG pathway gene sets. From each dataset, we extracted the top 10 significantly enriched pathways for each target gene, with an adjusted P‑value < 0.05 considered statistically significant. By comparing the enrichment results between the two diseases, we identified biological processes and functional associations—whether shared across diseases or specific to each—in which the relevant genes were enriched.

### 2.9 Immune cell infiltration

Using the CIBERSORT deconvolution algorithm with the LM22 leukocyte gene signature matrix, we estimated the relative proportions of 22 infiltrating immune cell types based on gene expression profiles to evaluate the immune microenvironment in the disease tissues. Given the limited sample size of the IgG4‑RD dataset, which compromises the statistical power of CIBERSORT, we additionally applied single‑sample gene set enrichment analysis (ssGSEA) to assess immune infiltration in this cohort. ssGSEA calculates enrichment scores for specific immune cell marker gene sets in individual samples, reflecting the relative infiltration levels of various immune populations, and is less stringent in sample size requirements, making it suitable for small‑sample datasets. To cross‑validate and comprehensively compare the immune profiles of the two diseases, we further applied the MCPcounter algorithm to both datasets to calculate the relative abundances of immune and stromal cells, based on the expression of cell‑type‑specific marker genes. This algorithm provides robust estimates across different platforms and sample sizes. Finally, Spearman’s correlation analysis was performed in each dataset to examine the associations between the candidate genes and the abundances of various immune cell types. To account for multiple comparisons, all P-values from the correlation analyses were adjusted using the Benjamini–Hochberg (BH) method to control the false discovery rate (FDR), with P < 0.05 considered statistically significant.

### 2.10 Construction of TF-mRNA-miRNA regulatory network

TRRUST (Transcriptional Regulatory Relationships Unraveled by Sentence-based Text Mining; http://www.grnpedia.org/trrust) is a manually curated repository of transcriptional regulatory networks. miRWalk (http://mirwalk.umm.uni-heidelberg.de) is a comprehensive database that curates predicted and experimentally validated microRNA (miRNA)–target gene interactions. To further elucidate the regulatory mechanisms governing the candidate genes, we retrieved their corresponding transcription factors (TFs) from the TRRUST database and predicted miRNA–mRNA interactions using the miRWalk platform. Finally, Cytoscape was used to construct TF- mRNA - miRNA regulatory networks.

## 3. Results

### 3.1 Identification of DEGs in the ANCA-GN and IgG4-RD

**Fig 2:**
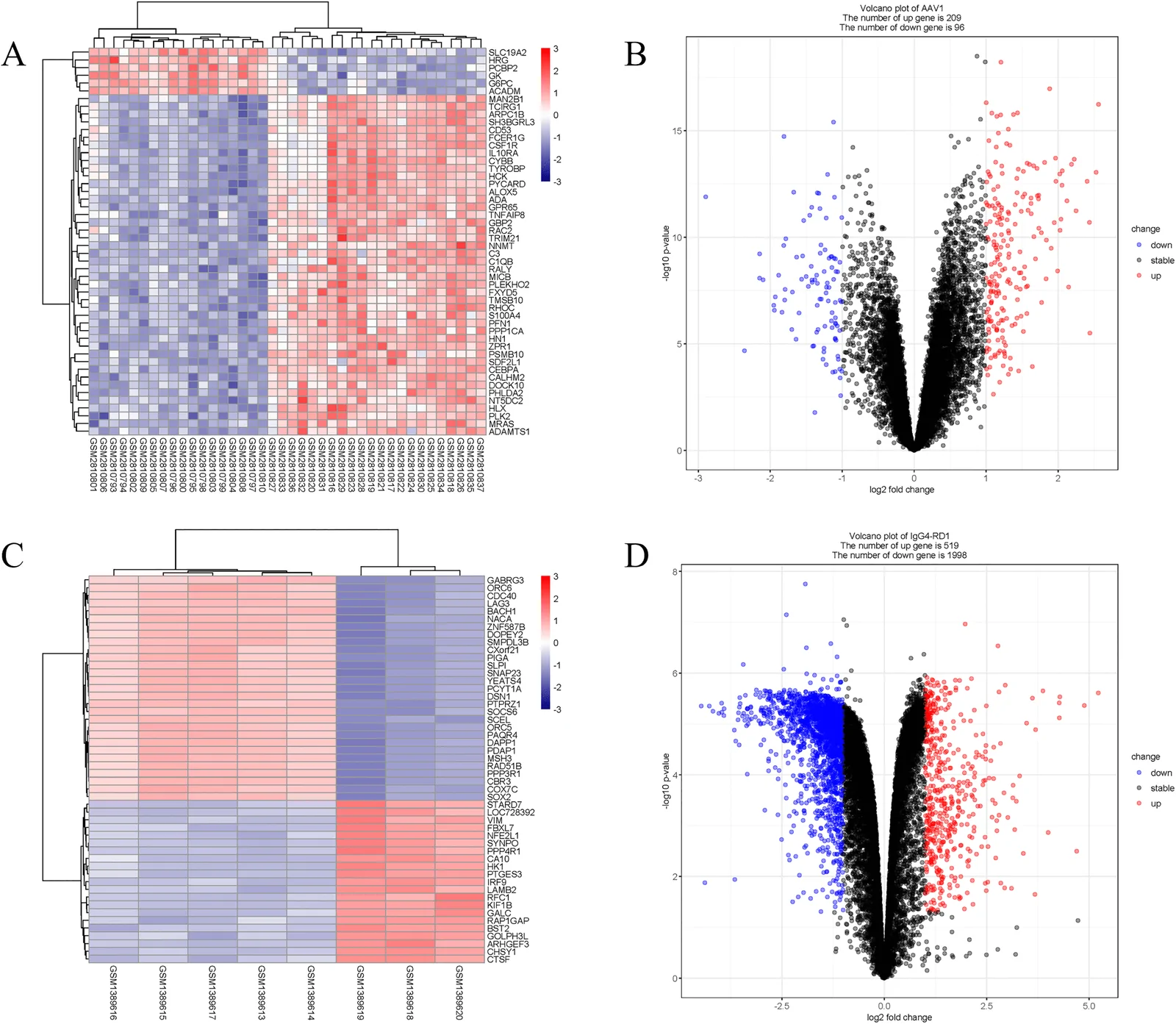
Analysis of DEGs. (A) Heatmap of top 50 DEGs in GSE104948 (red=up, blue=down). (B) Volcano plot: 209 upregulated (red) and 96 downregulated (blue) genes in ANCA‑GN. (C) Heatmap for IgG4‑RD (GSE40568). (D) Volcano plot: 519 up, 1998 down in IgG4‑RD. DEGs defined by adj.P < 0.05 and |log_2_FC| > 1.

After normalizing the data and correcting for batch effects using SVA, 305 differentially expressed genes (DEGs) were identified in the ANCA-GN dataset GSE104948, comprising 209 upregulated genes and 96 downregulated genes(Fig 2B). In the IgG4-RD dataset GSE40568, 2517 DEGs were identified, including 519 upregulated genes and 1998 downregulated genes(Fig 2D). Heatmaps displayed the expression levels of the top 50 genes across samples, while volcano plots visually represented the number and magnitude of differentially expressed genes(Fig 2A and Fig 2C). Since the IgG4‑RD dataset (GSE40568) was used solely for trend validation rather than for differential gene screening, Venn diagram analysis was not performed.

### 3.2 Enrichment analysis

**Fig 3:**
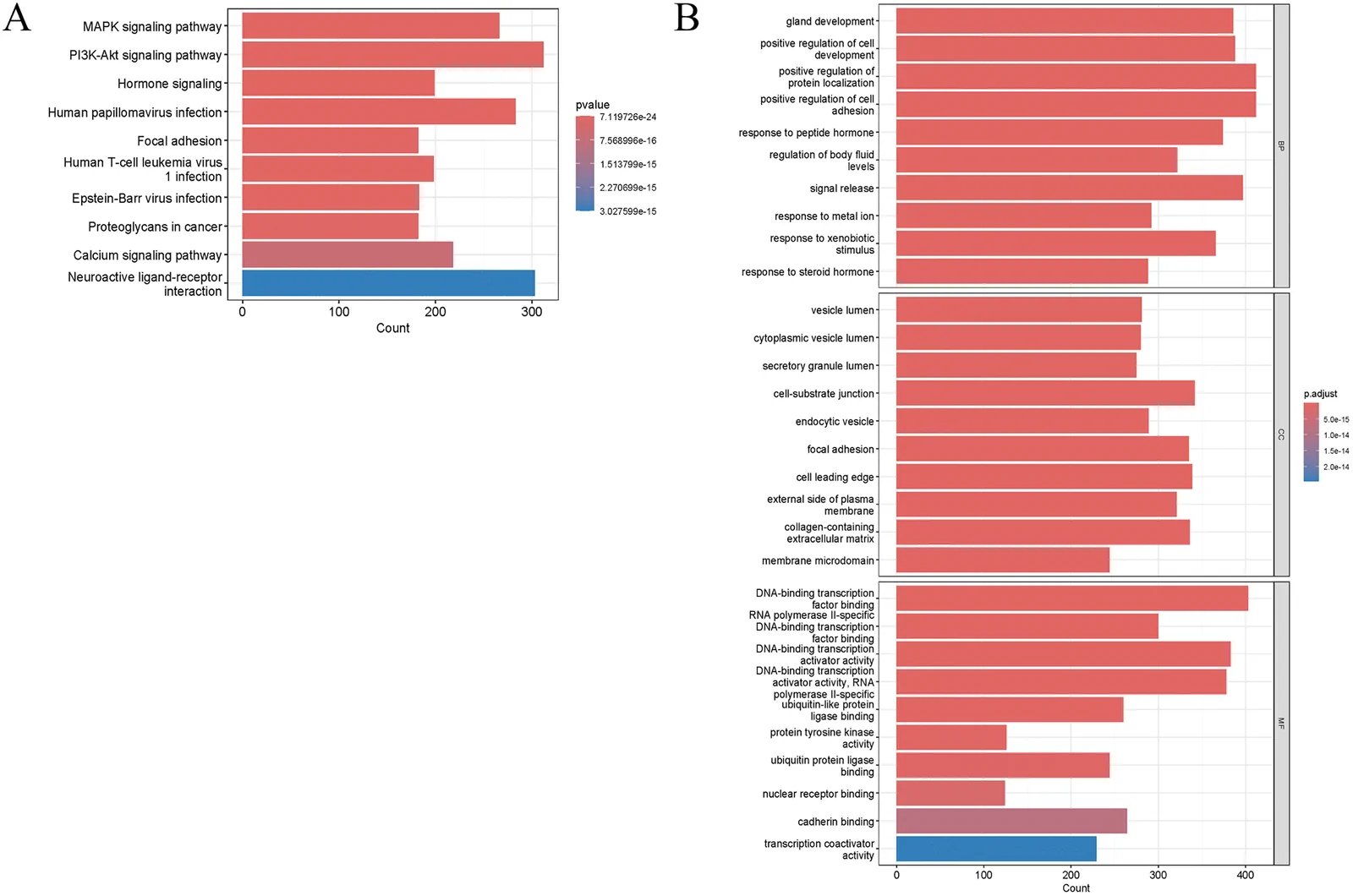
Enrichment analysis of DEGs. (A) KEGG bar plot showing top 10 pathways, including PI3K‑Akt, MAPK, calcium signalling, and viral infections. (B) GO analysis (BP, CC, MF) highlighting cell adhesion, secretory granules, and transcription factor binding. All adj.P < 0.05.

GO enrichment analysis (Fig 3B) revealed that the upregulated DEGs in the ANCA‑GN dataset were predominantly involved in biological processes including gland development, positive regulation of cell development, positive regulation of protein localization, positive regulation of cell adhesion, response to peptide hormone, regulation of body fluid levels, signal release, response to metal ion, response to xenobiotic stimulus, and response to steroid hormone. In terms of molecular function, these genes were enriched in DNA‑binding transcription factor binding, RNA polymerase II‑specific DNA‑binding transcription factor binding, DNA‑binding transcription activator activity, DNA‑binding transcription activator activity, RNA polymerase II‑specific, ubiquitin‑like protein ligase binding, ubiquitin protein ligase binding, protein tyrosine kinase activity, nuclear receptor binding, cadherin binding, and transcription coactivator activity. With respect to cellular components, the associated genes were primarily localized to vesicle lumen, cytoplasmic vesicle lumen, secretory granule lumen, cell‑substrate junction, endocytic vesicle, focal adhesions, cell leading edge, external side of plasma membrane, collagen‑containing extracellular matrix, and membrane microdomain. KEGG pathway analysis (Fig 3A) further indicated that these genes were significantly enriched in the PI3K‑Akt signaling pathway, neuroactive ligand–receptor interaction, human papillomavirus infection, the MAPK signaling pathway, calcium signaling, hormone signal transduction, human T‑cell leukemia virus type 1 infection, Epstein–Barr virus infection, focal adhesion, and proteoglycans in cancer.

### 3.3 PPI network

**Fig 4:**
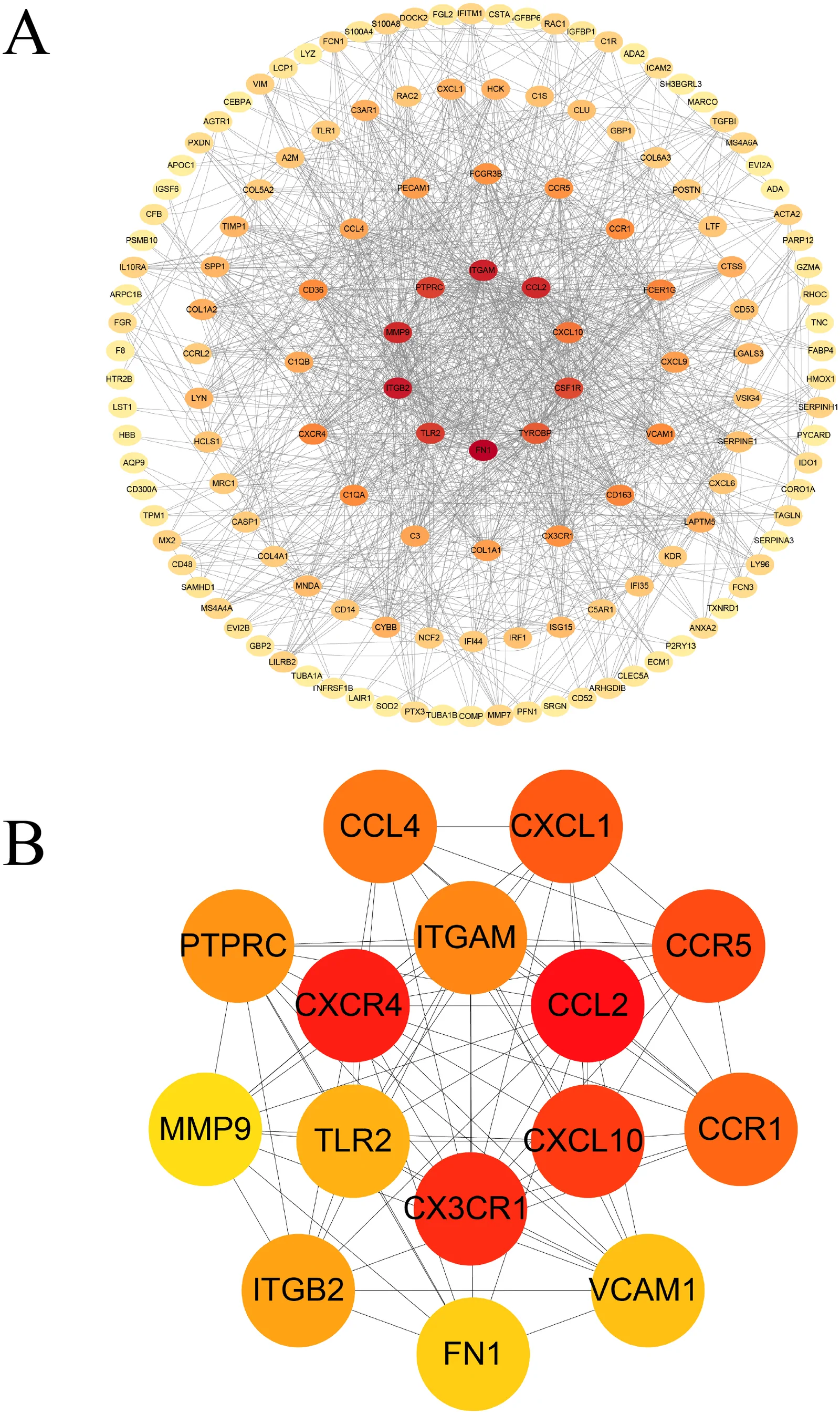
PPI network analysis. (A) Initial STRING network (confidence≥0.7) of 209 proteins. (B) Filtered network showing top 15 hub genes (red for higher MCC score, orange to yellow for lower). Selected genes include ITGAM, CXCL1, FN1, etc. Gene symbols follow HGNC nomenclature (ITGAM = integrin αM, CXCL1 = C‑X‑C motif chemokine ligand 1). Nodes = proteins, edges = interactions.

To investigate interactions among DEGs, we constructed a PPI network for 209 DEGs using the STRING online platform (Fig 4A). After removing isolated nodes, the network was imported into Cytoscape, yielding a PPI network diagram with 15 nodes and 79 edges. Using the MCC algorithm, we identified the top 15 genes including CCR5, CCR1, CXCL10, VCAM1, FN1, CX3CR1, ITGB2, TLR2, MMP9, CXCR4, PTPRC, ITGAM, CCL4, CXCL1, and CCL2 (Fig 4B).

### 3.4 Machine learning screening and validation of candidate genes

**Fig 5:**
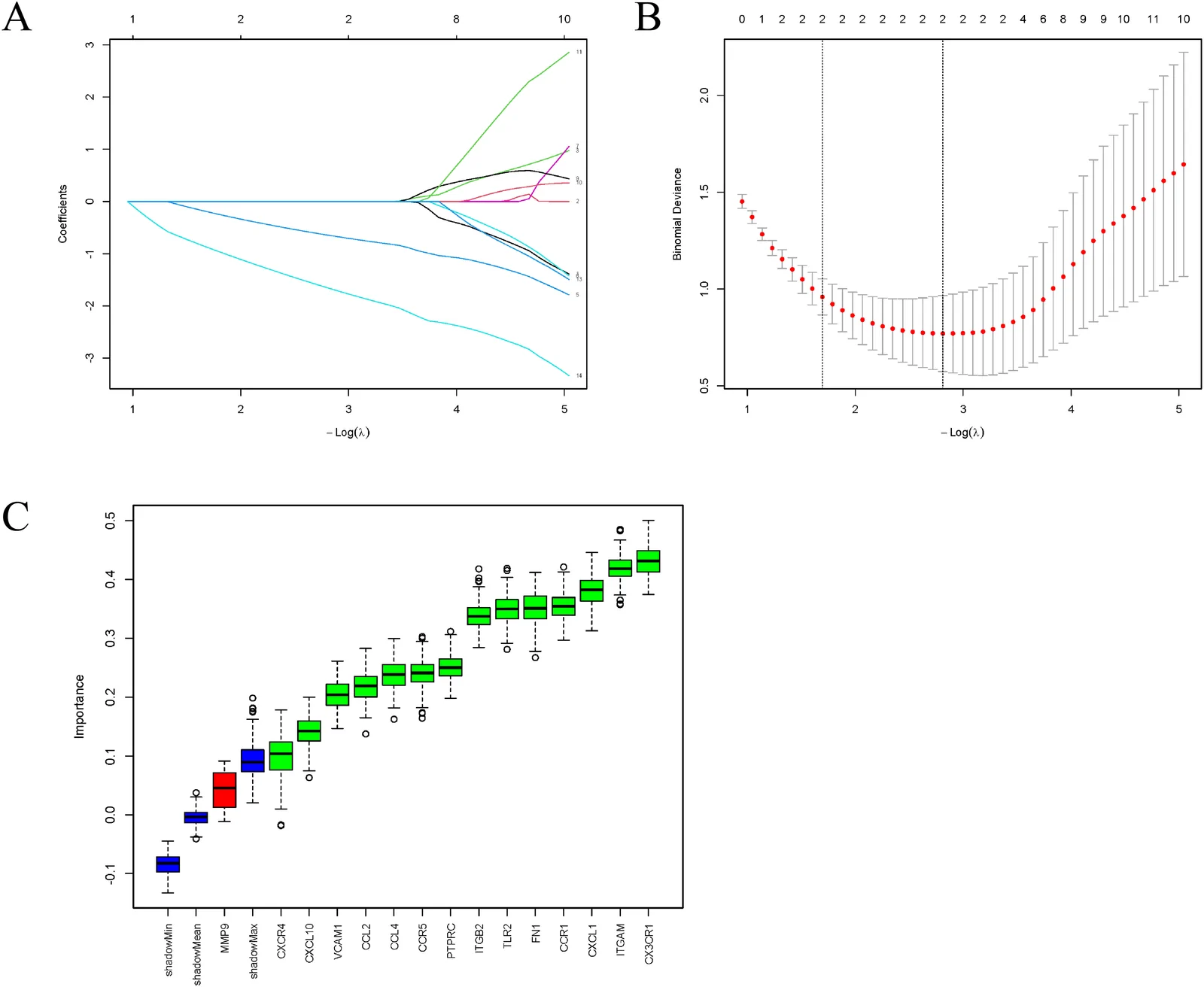
Lasso regression analysis and Boruta algorithm of candidate DEGs. (A) LASSO coefficient paths; (B) 10‑fold cross‑validation error vs. λ; (C) Boruta importance (green = confirmed, blue = shadow, red = rejected). Overlap of both methods yielded FN1, ITGAM, and CXCL1.

Due to the limited sample size of the IgG4‑RD dataset, which increases the risk of overfitting and compromises statistical power, machine learning‑based screening and validation were performed exclusively on the ANCA‑GN dataset. Lasso regression and the Boruta algorithm were applied to screen for candidate feature genes (Fig 5A, Fig 5B, Fig 5C). Lasso regression identified genes strongly associated with the disease, and the Boruta algorithm was subsequently used to validate the significance of these genes. Through this combined approach, three genes, FN1, ITGAM and CXCL1, were ultimately identified (Table 1).

**Table 1:** Comparison of machine learning results.

	Lasso regression analysis	Boruta algorithm
**ANCA**	VCAM1, FN1, ITGAM,CXCL1	CCR5 CCR1 FN1 CX3CR1 ITGB2 TLR2 MMP9 PTPRC ITGAM CXCL1

### 3.5 Validation of expression trends in IgG4‑RD

We mapped the three machine learning‑derived candidate core genes from ANCA‑GN to the differential expression data of IgG4‑RD and validated their upregulation consistency using a log_2_FC > 0 cutoff. Two genes, ITGAM (log_2_FC = 1.0409) and CXCL1 (log_2_FC = 0.399), showed positive log_2_FC values in IgG4‑RD, matching the upregulation pattern seen in ANCA‑GN. Thus, these two genes exhibit a shared upregulation trend across diseases and represent promising candidates for exploring common pathogenic mechanisms in cross‑tissue comorbidity.

### 3.6 Validation of candidate genes

**Fig 6:**
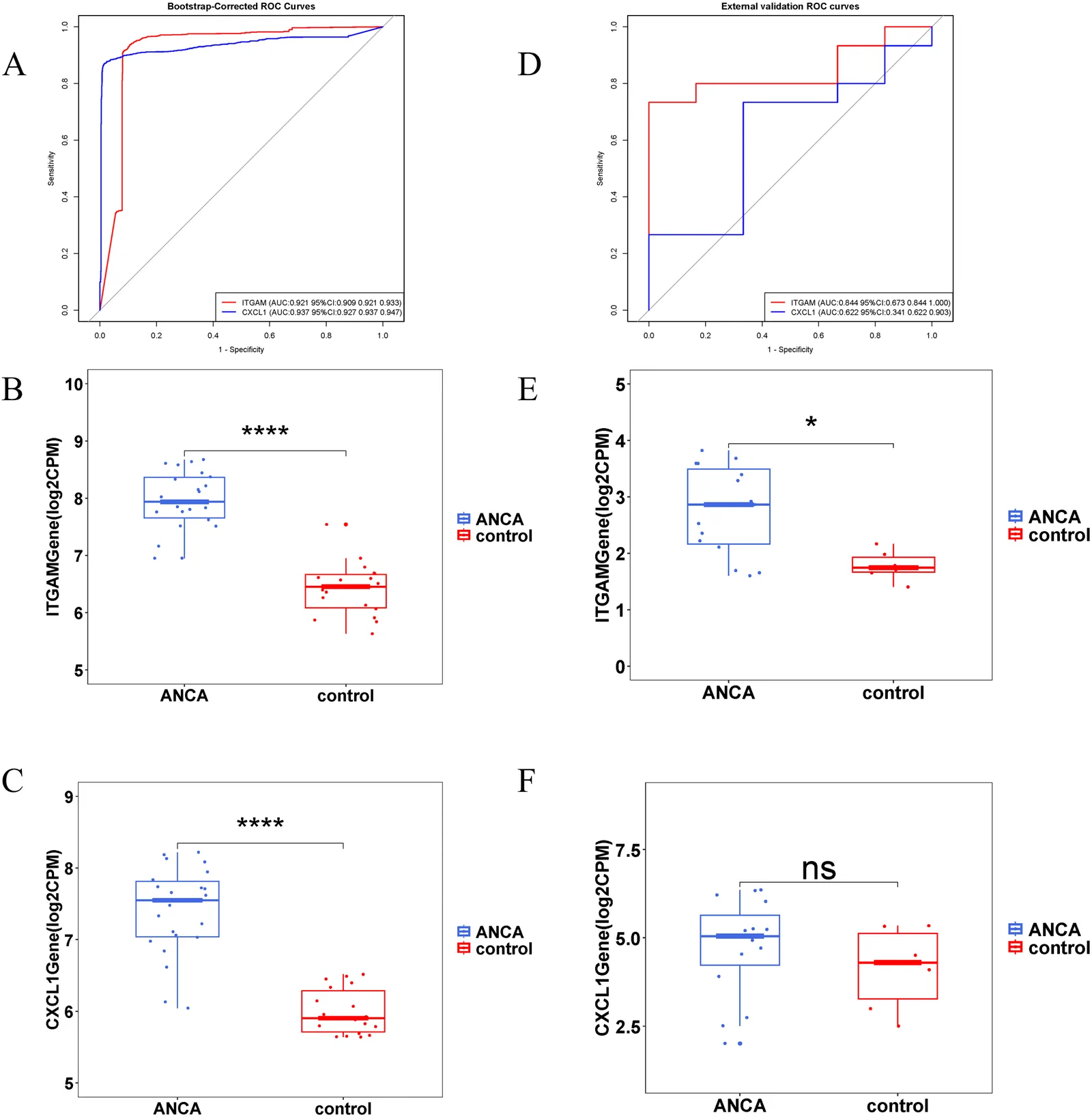
ROC curves. (A) ROC curves for ITGAM and CXCL1 after Bootstrap internal validation: ITGAM (AUC = 0.921) and CXCL1 (AUC = 0.937) show good predictive value. (B) Box plot of ITGAM expression in the internal cohort; (C) Box plot of CXCL1 expression in the internal cohort; (D) ROC curves for ITGAM and CXCL1 in the external validation cohort; (E) Box plot of ITGAM expression in the external validation cohort; (F) Box plot of CXCL1 expression in the external validation cohort.

To evaluate the predictive performance of the aforementioned candidate genes, we first performed bootstrap internal validation on the ANCA‑GN dataset GSE104948. The AUC for ITGAM was 0.921 (95% CI: 0.909 0.921 0.933), and that for CXCL1 was 0.937 (95% CI: 0.927 0.937 0.947)(Fig 6 A, Fig 6B, Fig 6C). In a subsequent external validation using the independent ANCA‑GN dataset GSE108109, ITGAM maintained good discriminatory power (AUC = 0.844, 95% CI: 0.673 0.844 1.000), whereas the AUC for CXCL1 dropped to 0.622 (95% CI: 0.341 0.622 0.903), suggesting limited predictive ability, possibly attributable to the small sample size of the external validation cohort and heterogeneity across different datasets(Fig 6D). In contrast, box plot analysis of the external validation cohort revealed no significant difference in CXCL1 expression between ANCA‑GN patients and healthy controls. This finding is consistent with the reduced AUC observed for CXCL1(Fig 6E, Fig 6F).

### 3.7 Gene set enrichment analysis of candidate genes

To further elucidate the biological functions of the candidate genes, we performed single‑gene GSEA for ITGAM and CXCL1 in both the ANCA‑GN and IgG4‑RD datasets, and extracted the top 10 significantly enriched pathways (adjusted P < 0.05) for each gene.

**Fig 7:**
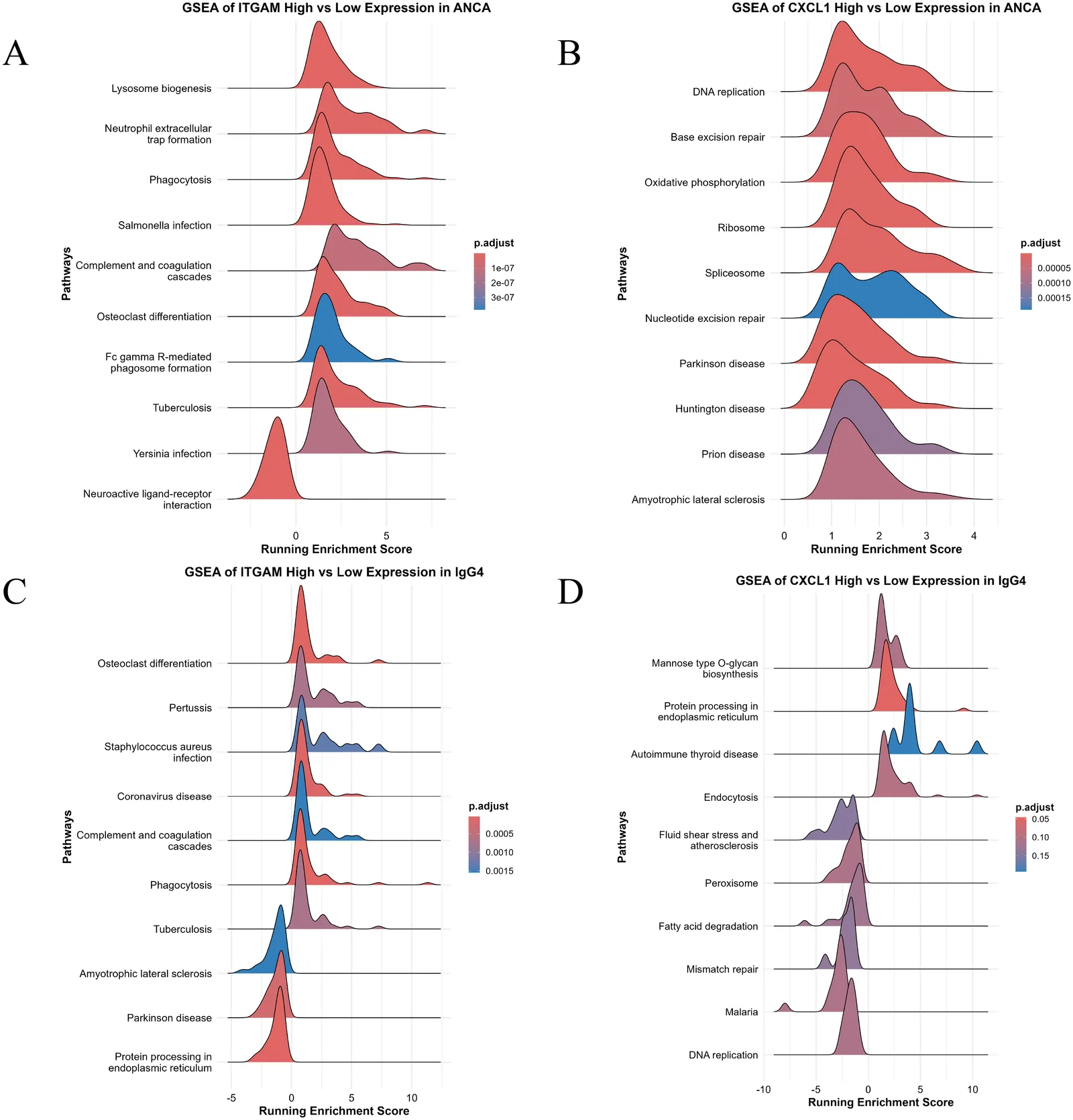
GSEA. (A) ITGAM in ANCA‑GN: positively enriched in phagocytosis, NET formation, complement cascades; negative in neuroactive ligand‑receptor. (B) CXCL1 in ANCA‑GN: positive in DNA replication, oxidative phosphorylation; negative in cytokine‑cytokine receptor. (C) ITGAM in IgG4‑RD: positive in phagocytosis, osteoclast differentiation, tuberculosis; negative in ER protein processing. (D) CXCL1 in IgG4‑RD: positive in ER processing, endocytosis; negative in DNA replication and atherosclerosis. All FDR < 0.05.

In the ANCA‑GN dataset (Fig 7A, Fig 7B), ITGAM showed positive enrichment in Lysosome, Phagocytosis, Salmonella infection, Neutrophil extracellular trap formation, Osteoclast differentiation, Tuberculosis, Complement and coagulation cascades, Yersinia infection, and Fc gamma R‑mediated phagosome formation, whereas negative enrichment was observed for Neuroactive ligand‑receptor interaction. CXCL1 was positively enriched in DNA replication, Oxidative phosphorylation, Parkinson disease, Huntington disease, Ribosome, Spliceosome, Base excision repair, Amyotrophic lateral sclerosis, Prion disease, and Nucleotide excision repair, with negative enrichment in Neuroactive ligand‑receptor interaction and Cytokine‑cytokine receptor interaction.

In the IgG4‑RD dataset (Fig 7C, Fig 7D), the enrichment profiles of the two genes shared both similarities and differences compared with those in ANCA‑GN. ITGAM was positively enriched in Osteoclast differentiation, Phagocytosis, Coronavirus disease, Pertussis, Tuberculosis, Staphylococcus aureus infection, and Complement and coagulation cascades, and negatively enriched in Protein processing in endoplasmic reticulum, Parkinson disease, and Amyotrophic lateral sclerosis. CXCL1 showed positive enrichment in Protein processing in endoplasmic reticulum, Endocytosis, Mannose type O‑glycan biosynthesis, and Autoimmune thyroid disease, with negative enrichment in DNA replication, Malaria, Peroxisome, Fatty acid degradation, Mismatch repair, Fluid shear stress and atherosclerosis, and Atherosclerosis.

Comparison of the enrichment results between the two datasets revealed that the candidate genes were involved, to varying degrees, in infection‑, immune‑, and metabolism‑related pathways in both diseases, while also highlighting some disease‑specific pathway associations.

### 3.8 Immune infiltration analysis

Both ANCA‑GN and IgG4‑RD are autoimmune inflammatory diseases where the inflammatory microenvironment alters immune cell distribution. To clarify the immune landscape, we performed infiltration analyses separately on the two datasets.

**Fig 8:**
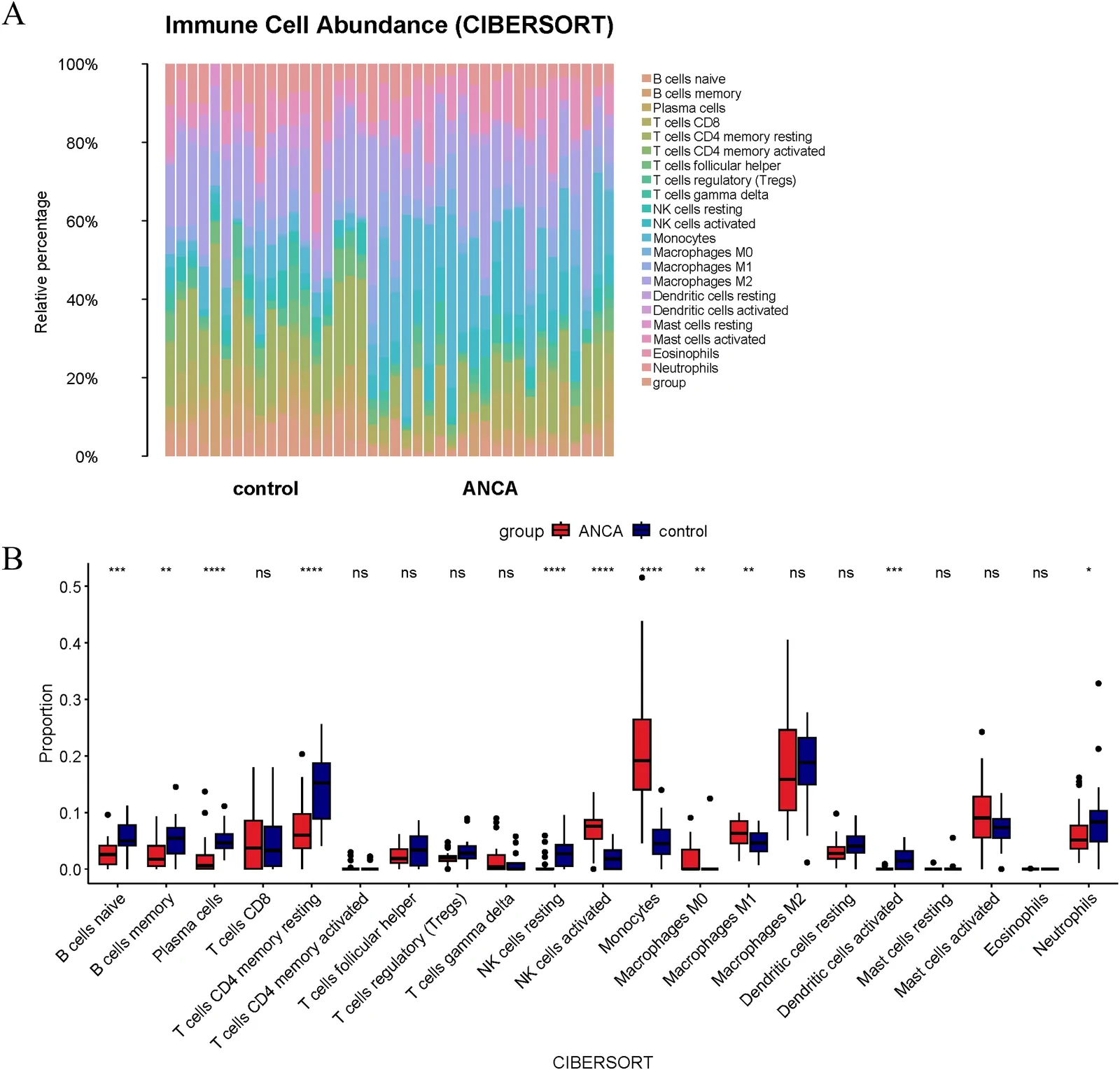
CIBERSORT immune infiltration analysis of ANCA-GN. (A B) CIBERSORT for ANCA-GN.

**Fig 9:**
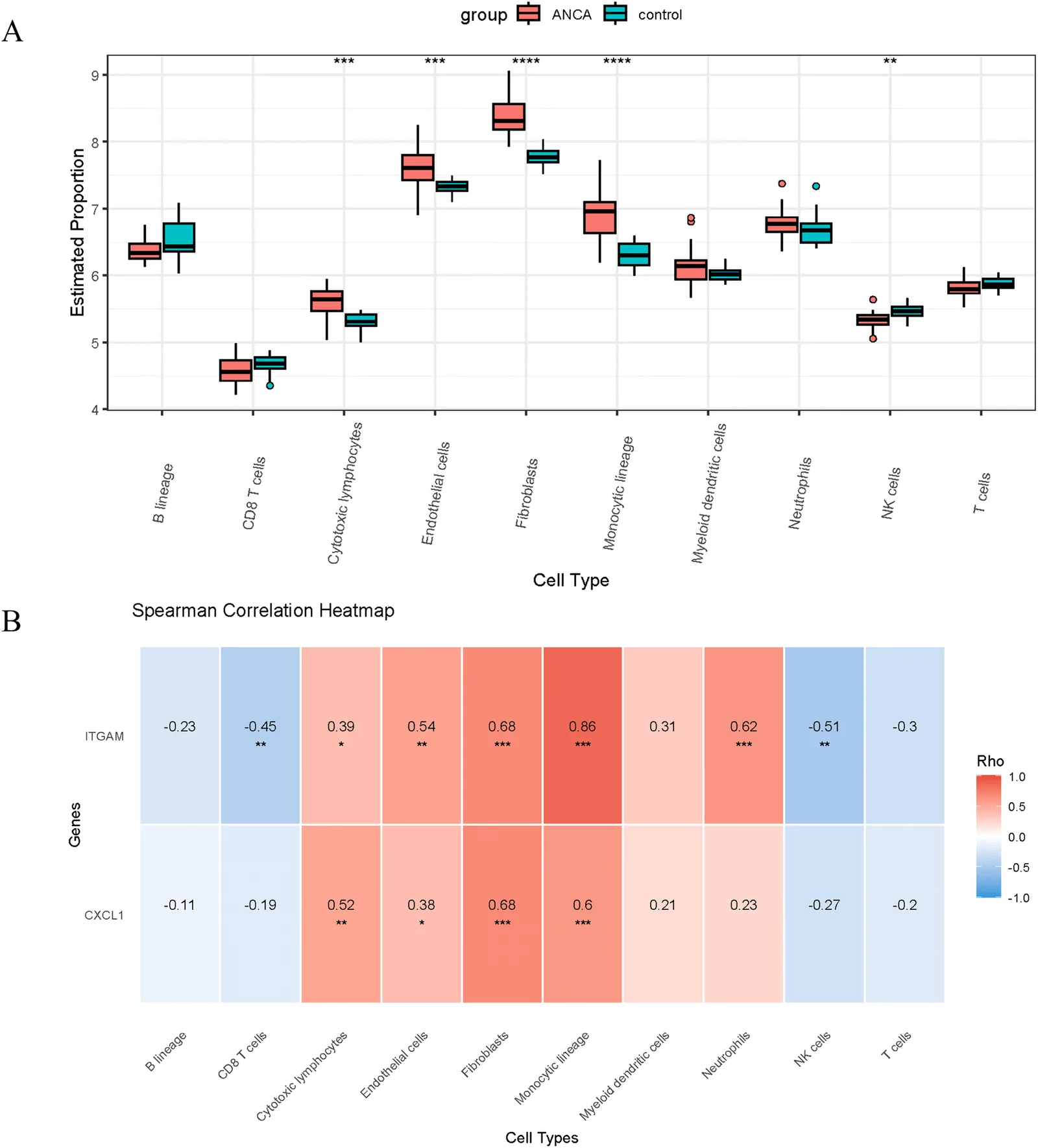
MCPcounter immune infiltration analysis and spearman correlation analysis of ANCA-GN. (A) MCPcounter for ANCA-GN. (B) Spearman Correlation Analysis for ANCA-GN.

In ANCA‑GN, we used CIBERSORT to generate stacked bar charts and box plots for intergroup comparisons of immune cell fractions, and employed MCPcounter for cross‑validation of stromal and immune abundances (Fig 8A, Fig 8B, Fig 9A). CIBERSORT showed elevated B cells, T cells, and monocytes, with decreased NK cells and M0 macrophages. MCPcounter conversely indicated downregulation of B‑lineage, CD8 ⁺ T, NK, and T cells, but upregulation of cytotoxic lymphocytes, monocytic lineage, myeloid dendritic cells, fibroblasts, neutrophils, and endothelial cells.

**Fig 10:**
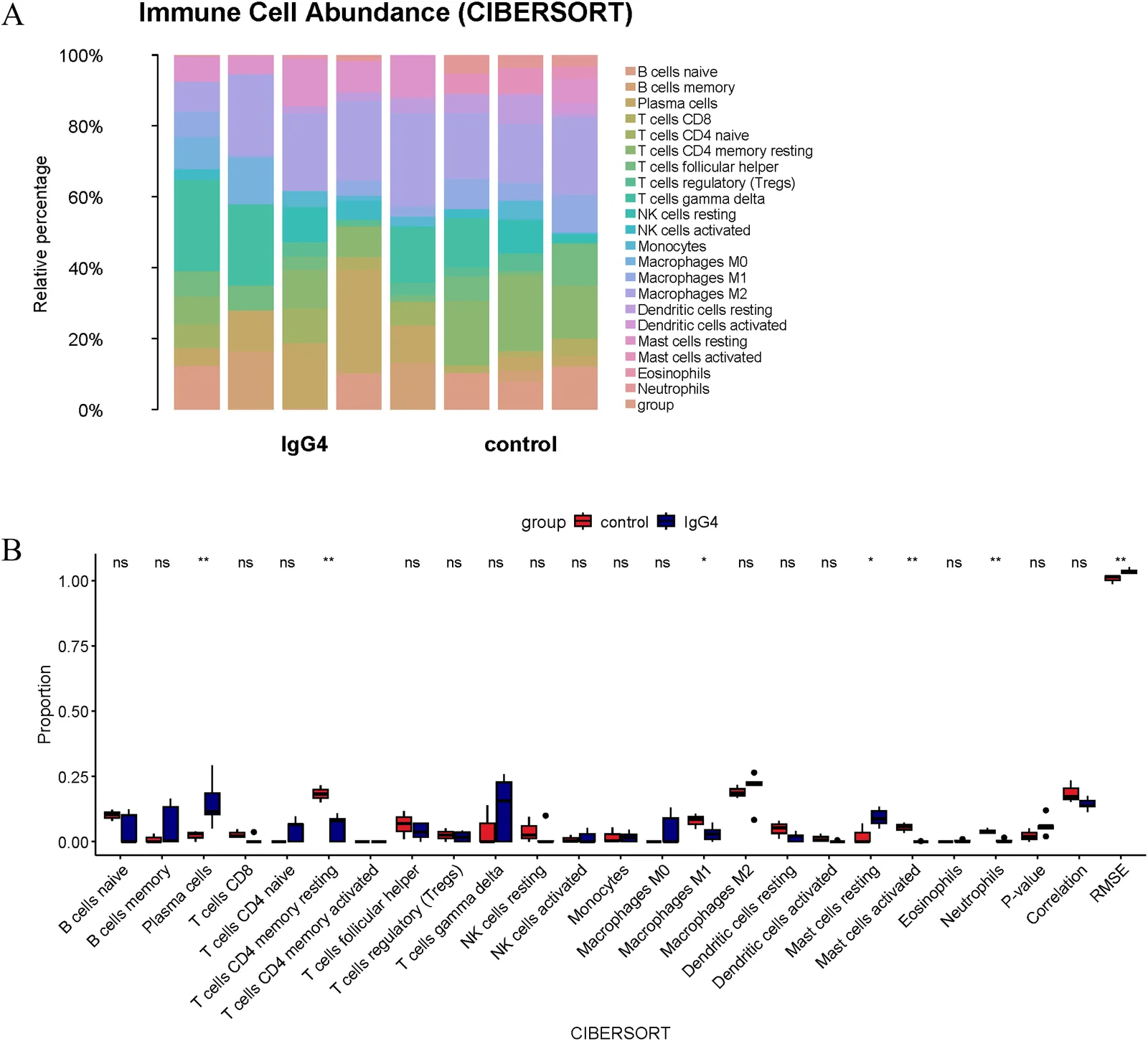
CIBERSORT immune infiltration analysis of IgG4-RD. (A B) CIBERSORT for IgG4‑RD.

**Fig 11:**
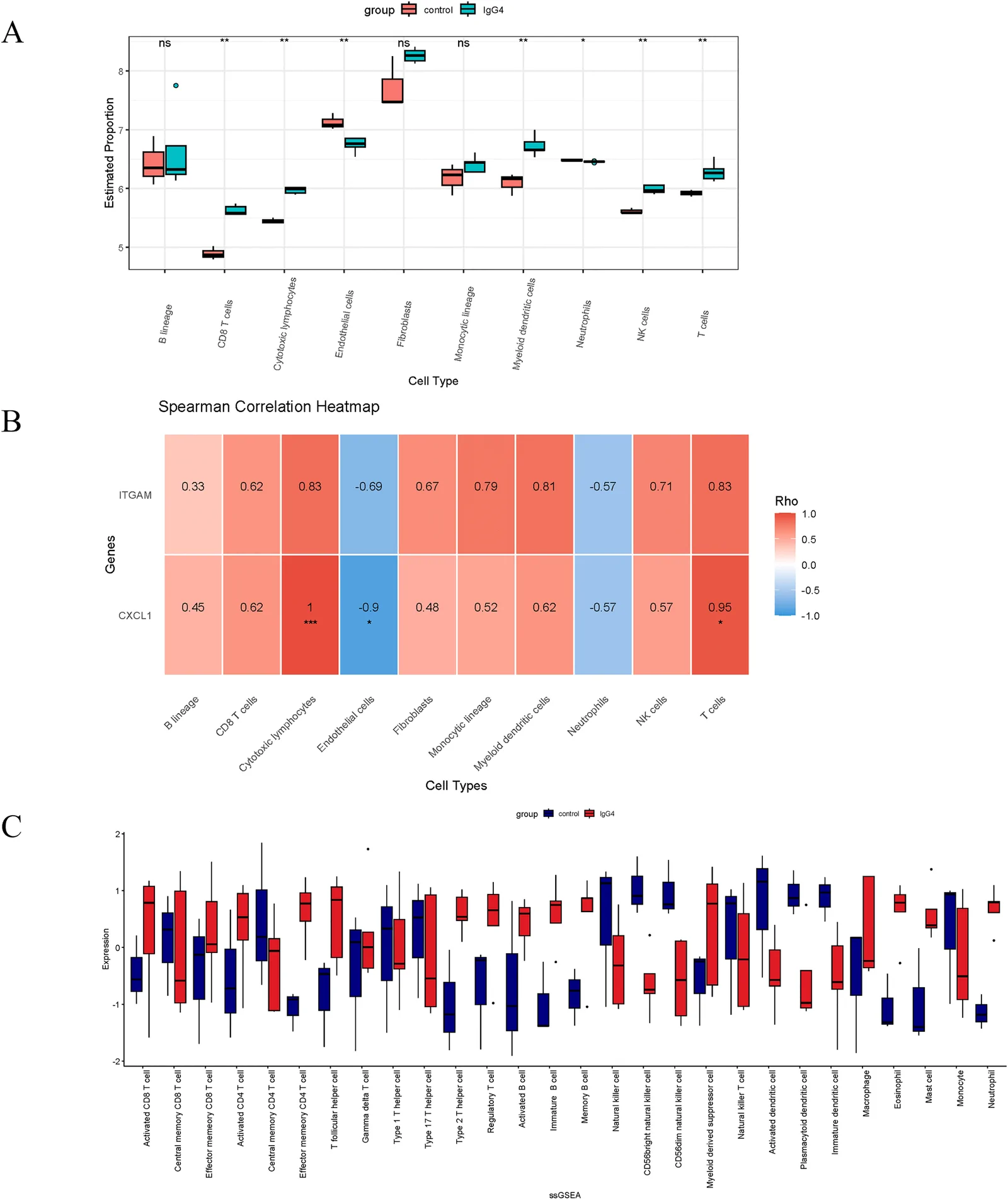
MCPcounter immune infiltration analysis and spearman correlation analysis of IgG4-RD. (A) MCPcounter for IgG4‑RD. (B) Spearman Correlation Analysis for IgG4‑RD. (C) ssGSEA for IgG4‑RD. * represents a p-value < 0.05; ** represents a p-value < 0.01; *** represents a p-value < 0.001; **** represents a p-value < 0.0001.

In IgG4‑RD, due to small sample size, we applied ssGSEA alongside CIBERSORT to estimate immune enrichment scores (Fig 10A, Fig 10B, Fig 11A, Fig 11C). CIBERSORT revealed higher plasma cells, resting mast cells, and M2 macrophages, and lower resting CD4 ⁺ memory T cells, M1 macrophages, activated mast cells, and neutrophils. Though ssGSEA P‑values were not significant (P > 0.05), trend analysis suggested increased activated, immature, and memory B cells, with decreased plasmacytoid dendritic cells. MCPcounter further validated these trends, showing downregulated endothelial cells and neutrophils, and upregulated T, CD8 ⁺ T, cytotoxic lymphocyte, NK, monocytic, myeloid dendritic, and fibroblast populations.

Spearman correlation analysis (Fig 9B, Fig 11B) revealed that in ANCA‑GN, after FDR correction, ITGAM and CXCL1 both correlated positively with cytotoxic lymphocytes, endothelial cells, neutrophils, monocytic lineage, and fibroblasts, and negatively with CD8 ⁺ T and NK cells (adjusted P < 0.05); ITGAM showed a stronger neutrophil association. In IgG4‑RD, following FDR correction, CXCL1 correlated positively with T and cytotoxic lymphocyte, but negatively with endothelial cells (adjusted P < 0.05). After correction, the correlations of ITGAM with cytotoxic lymphocytes, monocytic lineage, myeloid dendritic cells, and T cells were not statistically significant, but they still exhibited directional trends consistent with those of CXCL1. The correlations of the two genes with neutrophils, NK cells, T cells, CD8 ⁺ T cells, and endothelial cells were opposite to those observed in ANCA‑GN.

### 3.9 Construction of the TF–mRNA–miRNA regulatory network

**Fig 12:**
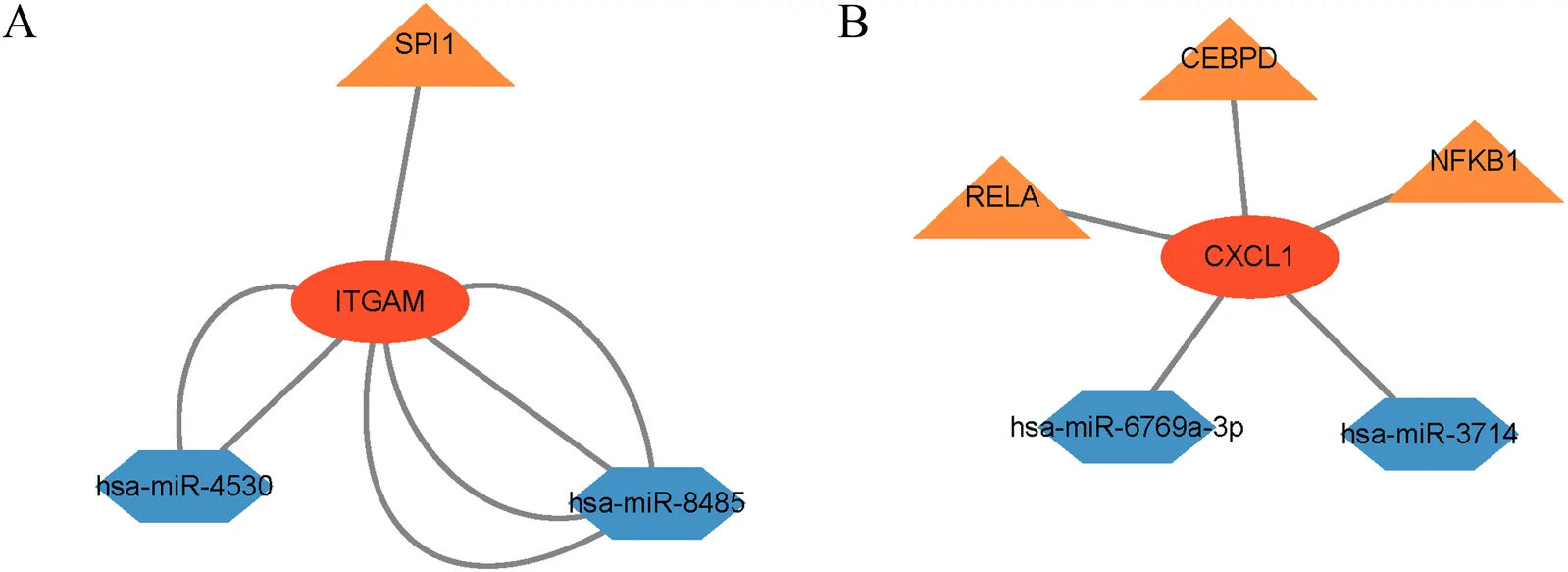
TF–mRNA–miRNA regulatory network. (A) ITGAM network: red = ITGAM, blue = TF SPI1, green = miRNAs (hsa‑miR‑4530, hsa‑miR‑8485). (B) CXCL1 network: red = CXCL1, blue = TFs (CEBPD, NFKB1, RELA), green = miRNAs (hsa‑miR‑3714, hsa‑miR‑6769a‑3p). Edges indicate regulatory relationships.

Based on the TRRUST and miRWalk databases, we identified four transcription factors (TFs) and five microRNAs (miRNAs) that were predicted to regulate the expression of the candidate genes. We then constructed a TF–mRNA–miRNA regulatory network using Cytoscape software (Fig 12A, Fig 12B).

## 4. Discussion

AAV is an autoimmune disease primarily causing inflammation of small blood vessels. An epidemiological study found that the incidence rate of AAV is approximately 17.2 per million person-years, with a prevalence rate of 198.0 per million person-years [12]. ANCA-GN is a rapidly progressive glomerulonephritis induced by AAV, with pathological features primarily including segmental glomerular necrosis with crescent formation and minimal or absent immune complex deposition [13]. IgG4-RD is a multiorgan disorder characterized by tumor-like fibroinflammatory lesions in affected organs and associated functional impairment; its precise prevalence in the population remains unclear [14]. Although emerging evidence from case reports suggests a potential common pathway between ANCA‑GN and IgG4‑RD, the underlying molecular mechanisms remain largely unexplored. Bioinformatics analysis offers an effective approach for comprehensively understanding disease pathophysiology at the genetic level. Accordingly, this study employed bioinformatics methods to investigate cross‑tissue transcriptomic associations between ANCA‑GN and IgG4‑RD, with the goal of establishing a theoretical foundation for the discovery of shared biomarkers.

We first identified upregulated differentially expressed genes (DEGs) in ANCA‑GN and performed systematic functional enrichment analyses on these genes. GO enrichment analysis showed that the associated biological processes were primarily involved in positive regulation of cell adhesion and responses to hormones and exogenous stimuli; cellular components were significantly enriched in secretory granule compartments and focal adhesions; and molecular functions were dominated by transcription factor binding and tyrosine kinase activity. KEGG pathway analysis further revealed significant enrichment in the PI3K‑Akt and MAPK signaling pathways, as well as pathways related to viral infections.

During ANCA‑GN pathogenesis, ANCA‑activated neutrophils release reactive oxygen species (ROS), proteases via degranulation, and neutrophil extracellular traps (NETs), which contribute to glomerular basement membrane degradation and segmental capillary loop necrosis, thereby promoting crescent formation [15]. Infections, as major environmental triggers of ANCA‑GN, promote the release of inflammatory cytokines. Under inflammatory stimulation, target antigens (MPO or PR3) within neutrophils translocate to the cell surface, where they are recognized by the immune system and activate B cells to produce anti‑neutrophil cytoplasmic antibodies (ANCA) [16]. The enrichment of PI3K‑Akt and MAPK pathways suggests M1‑type macrophage polarization. Macrophages have been shown to participate in crescent formation in ANCA‑GN [17]. In parallel, secrete pro‑inflammatory factors such as TNF‑α, IL‑1β, and the chemokine CXCL1, which further recruit neutrophils, amplify the inflammatory response, inhibit podocyte autophagy, accelerate podocyte apoptosis, and promote the loss of slit diaphragm proteins such as nephrin, ultimately driving the development of proteinuria [18,19]. In addition, the enrichment of calcium signaling pathways points to its potential role in mediating neutrophil activation and degranulation, as elevated intracellular calcium is a critical trigger for the respiratory burst and release of proteases in response to ANCA stimulation [20,21]. This may further amplify the local inflammatory cascade and contribute to glomerular injury. In summary, the coordinated aberrant activation of these pathways collectively drives inflammatory amplification, podocyte injury, and glomerular microcirculatory impairment, culminating in segmental necrosis and renal dysfunction.

Although IgG4‑RD is classically characterized by chronic fibrosis and plasma cell infiltration, it also involves mechanisms such as PI3K‑Akt/MAPK signaling‑driven pro‑fibrotic macrophage polarization and aberrant B‑cell differentiation. This suggests that the two conditions share overlapping core inflammatory signaling networks, providing a basis for future investigations into common pathogenic pathways across distinct disease entities.

To identify candidate biomarkers of a shared pathogenic pathway between ANCA‑GN and IgG4‑RD, we first imported the upregulated differentially expressed genes (DEGs) from ANCA‑GN into the STRING database to construct a protein‑protein interaction (PPI) network. Candidate genes were then screened using the MCC algorithm in Cytoscape, and the top 15 candidates were retained. These genes were subsequently subjected to further screening using two machine learning algorithms, Lasso regression and the Boruta algorithm. Applying a threshold of log_2_FC > 0, we retained genes that also exhibited upregulation in the IgG4‑RD dataset, which ultimately yielded two cross‑disease candidate targets, ITGAM and CXCL1. Finally, we validated their predictive performance using ROC curves in the AAV dataset. However, despite its high internal AUC (0.937), CXCL1 showed a markedly lower AUC of 0.622 in the independent external validation. This discrepancy may be attributed to the high sensitivity of CXCL1 expression to the local microenvironment, as well as to inter‑cohort differences in patient subtypes (e.g., MPO‑/PR3‑ANCA), disease activity, or sample processing protocols. Consequently, CXCL1 may have weaker generalizability as a single biomarker compared with ITGAM, and further validation in larger, multicenter cohorts is warranted.

ITGAM (integrin subunit αM) is an integrin α‑chain protein encoded by a gene located on chromosome 16. ITGAM encodes the integrin αM chain, which pairs with the β2 chain (ITGB2) to form the heterodimeric integrin Mac‑1 (CD11b/CD18, αMβ2, or complement receptor 3). This complex mediates neutrophil adhesion to and migration across activated endothelial cells, as well as the phagocytosis of microorganisms [22].

In ANCA‑GN, ITGAM (CD11b) serves as a key adhesion molecule on the surface of neutrophils and mononuclear macrophages. GSEA enrichment analysis revealed that ITGAM is positively enriched in lysosomes, phagosomes, FcγR‑mediated phagocytosis, neutrophil extracellular trap (NET) formation, and the complement and coagulation cascades, suggesting its involvement in degranulation, respiratory burst, and NETosis following ANCA‑mediated neutrophil activation, which in turn leads to glomerular basement membrane degradation and segmental necrosis. Previous studies measuring activated CD11b on peripheral blood neutrophils, as well as soluble CD11b in urine and plasma, have demonstrated that surface CD11b levels on peripheral neutrophils are significantly elevated in patients with ANCA‑GN compared with healthy controls. Moreover, the degree of elevation correlates positively with disease activity (BVAS), inflammatory markers (hCRP), renal function (serum creatinine), and the proportion of cellular crescents in renal biopsies, and is consistent with CD11b deposition in urine and renal tissue [23]. These findings suggest that CD11b activation not only reflects systemic disease activity but also contributes to local renal inflammatory damage, indicating its potential as a biomarker for monitoring ANCA‑GN and as a therapeutic target [24].

In IgG4‑RD, ITGAM is similarly enriched in phagocytic and complement pathways. Its ligands, ICAM‑1 and fibronectin, have been shown to be upregulated in various chronic fibrotic diseases [25,26]. Experimental evidence from hyperuricemia-related chronic kidney disease has shown that macrophage ITGAM can promote alternative macrophage polarization and renal fibrosis through FAK/AKT1/GSK3β signaling [27]. These observations suggest that ITGAM may contribute to IgG4‑RD pathogenesis by promoting M2 macrophage polarization, mediating chronic inflammation and phagocytic clearance, and participating in fibroblast activation and the initiation of fibrosis.

CXCL1 (C‑X‑C motif chemokine ligand 1, also known as melanoma growth‑stimulating activity factor α, MGSA‑α) is a secreted chemokine belonging to the CXC chemokine subfamily that mediates signal transduction through the G protein‑coupled receptor CXCR2 (CXC receptor 2) [28].

In ANCA‑GN, CXCL1 functions as a key CXC chemokine that recruits neutrophils and activates leukocytes, thereby playing a crucial role in inflammatory responses and angiogenesis [29]. Studies have demonstrated that neutralizing antibodies against CCL2 and CXCL1, or their respective receptor inhibitors, can suppress macrophage and neutrophil recruitment, reduce pro‑inflammatory cytokine expression and neutrophil infiltration in the kidneys, and alleviate renal inflammation [30,31].

GSEA enrichment analysis revealed that in ANCA‑GN, CXCL1 is positively enriched in pathways involved in DNA metabolism and protein synthesis, including DNA replication, ribosome, spliceosome, base excision repair, and nucleotide excision repair. This suggests a compensatory proliferative response of podocytes and parietal epithelial cells under sustained inflammatory stress, which may contribute to crescent formation. Additionally, CXCL1 is positively enriched in mitochondrial function‑related pathways, such as oxidative phosphorylation. Studies have found that circulating mtDNA levels are increased in patients with AAV and has been associated with disease activity. ANCA‑activated neutrophils undergo a burst of mitochondrial ROS production and release mitochondrial DNA, which forms neutrophil extracellular traps (NETs) that further exacerbate inflammation and tissue damage [32]. Moreover, mitochondrial dysfunction in renal tubular epithelial cells also contributes to tubular injury and interstitial fibrosis in ANCA‑GN.

A clinical study evaluating chemokine expression and its correlation with disease activity in patients with microscopic polyangiitis (MPA) and granulomatosis with polyangiitis (GPA) (MPA/GPA) found that CXCL1, CCL4, and CX3CL1 were associated with disease activity, with CXCL1 expression being higher in patients with a disease duration of less than three months and in ANCA‑positive patients [33]. Another study reported elevated CXCL1 levels in patients with active GPA, which were closely correlated with disease activity [34].

In IgG4‑RD, CXCL1 was positively enriched in protein processing in the endoplasmic reticulum and mannose type O‑glycan biosynthesis, suggesting its potential role as a paracrine signal involved in endoplasmic reticulum stress and secretory dysfunction in acinar epithelial cells. In contrast, its negative enrichment in DNA replication and mismatch repair is consistent with the chronic fibrotic features observed in salivary duct epithelium, including accumulation of autophagic vesicles, dense fiber bundles, reduced secretory granules, widened intercellular spaces, swollen mitochondria, and dilated endoplasmic reticulum [35].

In summary, while ITGAM and CXCL1 are commonly upregulated in both ANCA‑GN and IgG4‑RD, their pathological effects diverge according to local tissue context. In the immune‑active glomerular microenvironment, they promote acute necrosis, whereas in the hypovascular, secretory epithelium of the salivary glands, they drive chronic fibrosis. This distinction supports the rationale for investigating shared pathogenic pathways and discovering cross‑disease biomarkers.

Through immune infiltration analysis, we found that ITGAM and CXCL1 were significantly associated with multiple immune cell subsets in both ANCA‑GN and IgG4‑RD, although their effector cell profiles and pathological patterns differed between the two diseases.

In ANCA‑GN, CIBERSORT and MCPcounter revealed significantly elevated proportions of neutrophils, monocytic lineage cells, B cells, and T cells. Spearman’s correlation analysis further indicated that both ITGAM and CXCL1 were significantly positively correlated with neutrophils, endothelial cells, fibroblasts, and the monocytic lineage. Previous studies have shown that T cells accumulate in inflamed renal tissue in ANCA‑GN, and that Th cells and Tregs synergistically promote B‑cell differentiation into plasma cells, which produce ANCA. In addition, Tregs can downregulate anti‑MPO antibody production and ameliorate anti‑MPO‑induced glomerulonephritis [36]. Targeting T-cell-related immune checkpoints has been proposed as a potential therapeutic strategy for AAV and other autoimmune diseases [37].

In IgG4‑RD, immune infiltration in the salivary glands is characterized by elevated levels of plasma cells, resting mast cells, and M2‑type macrophages, along with significant upregulation of various B‑cell subsets. This profile is consistent with the classic pathological features of IgG4‑RD, including IgG4 ⁺ plasma cell‑rich infiltration, a Th2‑skewed immune response, and fibrosis. Correlation analysis revealed that ITGAM and CXCL1 were positively correlated with T cells, NK cells, dendritic cells, and fibroblasts, but negatively correlated with endothelial cells and neutrophils. These findings suggest that, within the chronic fibrotic microenvironment, both molecules participate in M2 polarization of monocytes/macrophages, mediate crosstalk between antigen‑presenting cells and lymphocytes, and contribute to fibroblast activation, thereby sustaining chronic inflammation and fibrotic progression. Studies have demonstrated that B cells from IgG4‑RD patients produce the pro‑fibrotic molecule PDGF‑B, which stimulates fibroblasts to produce collagen and induces the secretion of chemokines CCL4, CCL5, and CCL11, thereby promoting tissue fibrosis [38]. Furthermore, the robust clinical response to B‑cell depletion therapy and the identification of multiple autoantigens that drive B‑cell expansion underscore the central role of B cells in IgG4‑RD pathogenesis [39].

In summary, the immunological features of ITGAM and CXCL1 are highly consistent with the classic pathological hallmarks of ANCA‑GN (neutrophil‑mediated acute necrosis) and IgG4‑RD (plasma cell‑ and M2 macrophage‑mediated chronic fibrosis). This consistency provides a theoretical basis for exploring shared pathogenic pathways between the two diseases and for screening biomarkers from an immunological perspective. However, both ITGAM and CXCL1 have been previously reported to be closely associated with myeloid cells and inflammatory pathways, participating in processes such as inflammatory chemotaxis, neutrophil recruitment, and macrophage infiltration [40,41]. Given the distinctly different histological backgrounds of ANCA‑GN and IgG4‑RD, the concordant expression of these genes across the two diseases may partly reflect shared activation of myeloid‑related inflammatory pathways, rather than a disease‑specific cross‑tissue association. Consequently, these candidate genes may serve primarily as broad‑spectrum inflammatory markers, and their disease specificity warrants further evaluation through comparisons with other autoimmune conditions. Overall, the present findings are exploratory, and additional biochemical assays on blood or tissue samples from patients with concurrent diagnoses of both diseases are needed to further substantiate their predictive value.

To further elucidate the regulatory mechanisms of these two key candidate genes, we constructed a TF–mRNA–miRNA regulatory network. The regulatory network of ITGAM comprises one transcription factor, SPI1, and two microRNAs, hsa‑miR‑4530 and hsa‑miR‑8485. SPI1 encodes the PU.1 transcription factor, a master regulator of myeloid and B‑lymphocyte development [42], suggesting its potential regulatory role in ANCA‑GN.The regulatory network of CXCL1 consists of three transcription factors (CEBPD, NFKB1, and RELA) and two miRNAs (hsa‑miR‑3714 and hsa‑miR‑6769a‑3p). CEBPD is a key regulator of macrophage function and promotes TNF‑α‑stimulated inflammatory responses [43]. Both NFKB1 and RELA are members of the NF‑κB family and serve as central transcriptional regulators of inflammation, immunity, and stress responses; RELA (p65), in particular, is the key transcriptionally active subunit of the canonical NF‑κB pathway [44,45]. Collectively, these findings suggest that CXCL1 expression is driven by inflammatory signaling.Research on the roles of these transcription factors in ANCA‑GN and IgG4‑RD remains limited. Their regulatory interactions with target genes, as well as the underlying mechanisms in the context of these diseases, warrant further investigation and experimental validation.

## 5. Limitations

Several limitations of this study should be acknowledged. First, there is heterogeneity in tissue sources. This study is primarily based on bioinformatics analyses of public databases. Owing to the lack of kidney tissue datasets for IgG4‑related kidney disease (IgG4‑RKD), we used labial salivary gland tissue from patients with IgG4‑related sialadenitis as a substitute, which may have introduced tissue‑specific differences. Moreover, the candidate genes and their regulatory mechanisms have not yet been functionally validated through in vivo or in vitro experiments; therefore, the biological robustness of our conclusions requires further confirmation.

Second, the sample size of the IgG4‑RD dataset is limited, which severely constrains the statistical power of the differential expression analysis. This precluded formal differential screening and machine learning‑based validation on this dataset; we therefore relied solely on the direction of expression trends (log_2_FC sign) as the basis for evaluation. This approach may have introduced a risk of false positives. In addition, the immune infiltration analyses (e.g., ssGSEA) did not reach statistical significance and can only be interpreted as indicative trends.

Third, experimental validation is lacking. This study is entirely based on bioinformatics predictions derived from public databases. The expression levels, biological functions, and effects of targeted modulation of ITGAM and CXCL1 have not been validated in in vitro cell models or in vivo animal models. Furthermore, blood or tissue samples from patients with clinically confirmed diagnoses of both diseases have not been collected for qPCR, Western blotting, or immunohistochemical analyses. Consequently, the conclusions remain bioinformatics‑based hypotheses, and their clinical translational value awaits confirmation through experimental evidence.

Fourth, datasets from other autoimmune diseases were not included in the analysis. For instance, differences between MPO‑ANCA and PR3‑ANCA subtypes, heterogeneity across affected organs in IgG4‑RD, lupus nephritis, and IgA vasculitis‑associated nephritis were not examined. As a result, whether the expression of ITGAM and CXCL1 is influenced by disease subtypes or the spectrum of affected organs remains unknown.

In summary, the findings of this study should be considered exploratory. They provide candidate targets and a theoretical framework for future functional studies on the mechanisms underlying cross‑disease comorbidity.

## 6. Conclusions

Using bioinformatics and machine learning approaches, this study identified ITGAM as a potential shared biomarker for ANCA‑GN and IgG4‑RD, with robust predictive performance in both internal and external validation, whereas the poor external validity of CXCL1 indicates that its value requires further validation. However, these findings are entirely derived from bioinformatics analyses of public databases and should therefore be interpreted as hypothesis‑generating. Future studies are warranted to validate the expression and regulatory mechanisms of ITGAM and CXCL1 through multi‑level experiments, including in vitro cell models, in vivo animal models, and disease‑specific tissues with adequate sample sizes, using techniques such as qPCR, Western blotting, and immunohistochemistry. Such validation will be essential to assess their clinical translational value as cross‑disease biomarkers. In addition, further elucidation of the regulatory roles of transcription factors, including SPI1, CEBPD, NFKB1, and RELA, in modulating ITGAM and CXCL1 expression will help to unravel the molecular regulatory networks underlying the comorbidity of these two diseases and provide a theoretical basis for the development of targeted therapeutic strategies.
